# Dietary Supplementation with Epicatechin Improves Intestinal Barrier Integrity in Mice

**DOI:** 10.3390/foods11203301

**Published:** 2022-10-21

**Authors:** Jin Wan, Li Zhang, Zheng Ruan

**Affiliations:** 1 International Institute of Food Innovation, Nanchang University, Nanchang 330200, China; 2 College of Food Science and Technology, Nanchang University, Nanchang 330047, China

**Keywords:** epicatechin, antioxidant capacity, inflammatory responses, cell apoptosis, intestinal barrier

## Abstract

Epicatechin (EPI) is a dietary flavonoid that is present in many foods and possesses various bioactivities. We assessed the effects of EPI supplementation on intestinal barrier integrity in mice. Thirty-six mice were assigned to three groups and fed a standard diet or a standard diet supplemented with 50 or 100 mg EPI/kg (*n* = 12 per group). After 21 days of rearing, blood and intestinal samples were collected from eight randomly selected mice. Supplementation with 50 and 100 mg/kg EPI decreased (*p* < 0.05) the serum diamine oxidase activity and _D_-lactic acid concentration and increased (*p* < 0.05) the duodenal, jejunal, and ileal abundance of tight junction proteins, such as occludin. Moreover, it lowered (*p* < 0.05) the duodenal, jejunal, and ileal tumor necrosis factor-α contents and enhanced (*p* < 0.05) the duodenal and jejunal catalase activities and ileal superoxide dismutase activity. Supplementation with a lower dose (50 mg/kg) decreased (*p* < 0.05) the ileal interleukin-1β content, whereas supplementation with a higher dose (100 mg/kg) increased (*p* < 0.05) the duodenal and jejunal glutathione peroxidase activities. Furthermore, supplementation with 50 and 100 mg/kg EPI decreased (*p* < 0.05) cell apoptosis, cleaved cysteinyl aspartate-specific proteinase-3 (caspase-3), and cleaved caspase-9 contents in the duodenum, jejunum, and ileum. In conclusion, EPI could improve intestinal barrier integrity in mice, thereby suppressing intestinal inflammation and oxidative stress and reducing cell apoptosis.

## 1. Introduction

In addition to serving as the main site for nutrient digestion and absorption, the intestinal epithelium acts as a barrier to inhibit the passage of toxins, allergens, and pathogens from the luminal environment into the circulatory system [1,2]. However, numerous factors, such as inflammation and oxidative stress, can disrupt the intestinal barrier and thereby negatively affect health [3,4]. Therefore, dietary intervention with certain nutrients that can inhibit intestinal inflammation and oxidative stress is likely to attenuate intestinal barrier damage. Flavonoids are polyphenols containing diphenylpropane (C_6_-C_3_-C_6_); they are secondary metabolites that are ubiquitously distributed throughout the plant kingdom [5]. Recently, flavonoids have been a focus of research interest because of their potential modulatory properties with respect to intestinal permeability [6].

Epicatechin (EPI) is a polyphenolic flavonoid that belongs to the flavan-3-ol group; it is present in large amounts in tea, berries, and cocoa [7,8]. Importantly, EPI exhibits a wide array of physiological activities, such as anti-oxidative [9,10], anti-inflammatory [11,12], and anti-apoptotic [13,14] activities. Given these advantages, dietary EPI supplementation may be beneficial for the gastrointestinal tract. For instance, EPI has been reported to protect the gastrointestinal tract from the negative impacts of a high-fat diet [15,16]. However, to date, no study has clearly reported the effects of dietary EPI supplementation on intestinal barrier function in mice. Hence, further research is necessary to elucidate this.

Therefore, this study assesses the effects of EPI supplementation on intestinal barrier integrity in mice and explores its underlying mechanisms. Our findings would provide valuable information on dietary intervention with EPI for improving intestinal barrier function.

## 2. Materials and Methods

### 2.1. Animal Management and Diet

A total of 36 healthy mice (4 weeks old, weighing 24–30 g) were bought from Changsha Tianqin Biotechnology Co., Ltd. (Changsha, China) and acclimated for 1 week. Then, the mice were divided into three groups (*n* = 12 per group). The groups were randomly assigned to one of the three dietary treatments, namely a standard diet (control (CON) group) or a standard diet supplemented with 50 mg/kg EPI (LEPI group) or 100 mg/kg EPI (HEPI group) (purity >90%; Dalian Meilun Biotechnology Co., Ltd., Dalian, China). All the mice were individually caged under controlled conditions (temperature 24 ± 1 °C, humidity 50–60%, 12 h light–dark cycle) and provided with unlimited access to feed and autoclaved water during the 3-week experimental period.

### 2.2. Assessment of Weight Gain

On days 1 and 22 (08:00 h), all the mice were weighed to calculate their average daily gain (ADG) over the experimental period.

### 2.3. Sample Collection

After overnight fasting, eight randomly selected mice per group were anesthetized using ether (08:00 h on day 22) and their blood samples were collected via heart puncture. After being placed at room temperature for 30 min, the samples were centrifuged at 3000× *g* at 4 °C for 10 min to acquire serum and then stored at −20 °C. After blood sampling, the abdominal cavity was opened, and approximately 2 cm segments of the duodenum, jejunum, and ileum were fixed in 4% paraformaldehyde buffer for evaluating intestinal cell apoptosis. The samples were then flushed gently with ice-cold phosphate-buffered saline (PBS). Following this, mucosal samples from the duodenum, jejunum, and ileum were collected by scraping using a scalpel and frozen at −80 °C.

### 2.4. Measurement of Serum Parameters

Serum diamine oxidase (DAO) activity was measured using kits manufactured by Nanjing Jiancheng Bioengineering Institute (Nanjing, China), and serum _D_-lactic acid concentration was measured using ELISA kits manufactured by Jiangsu Meimian Industrial Co., Ltd. (Yancheng, China). All assays were performed according to the specific procedures provided in the kits.

### 2.5. Small Intestine Biochemical Analysis

#### 2.5.1. Preparation of Intestinal Homogenates

Frozen duodenal, jejunal, and ileal mucosal samples were thawed, mixed with ice-cold physiological saline at a ratio of 1:9 (*w*/*v*) and centrifuged for 10 min (3000× *g* at 4 °C) to obtain supernatants. Then, the total protein concentration in the supernatants was estimated using a total protein quantitative assay kit (Nanjing Jiancheng Bioengineering Institute). The antioxidant-related indices and cytokine and cell apoptosis-related protein contents were normalized against the total protein concentration in each sample for intersample comparison.

#### 2.5.2. Determination of Intestinal Antioxidant Capacity

Duodenal, jejunal, and ileal catalase (CAT), superoxide dismutase (SOD), and glutathione peroxidase (GSH-PX) activities and malondialdehyde (MDA) concentration were determined based on the manuals provided by Nanjing Jiancheng Bioengineering Institute.

#### 2.5.3. Assessment of Intestinal Cytokine and Cell Apoptosis-Related Protein Contents

Duodenal, jejunal, and ileal interleukin-1β (IL-1β), IL-10, tumor necrosis factor-α (TNF-α), interferon-γ (IFN-γ), B-cell lymphoma-2-associated X protein (Bax), B-cell lymphoma-2 (Bcl-2), TNF receptor 1 (TNFR1), cleaved cysteinyl aspartate-specific proteinase-3 (caspase-3), cleaved caspase-8, and cleaved caspase-9 contents were assessed using ELISA kits (Jiangsu Meimian Industrial Co., Ltd.). All operations were strictly manipulated according to the manufacturer’s guidelines.

### 2.6. Detection of Intestinal Cell Apoptosis

Apoptosis was assessed by a terminal deoxynucleotidyl transferase (TdT)-mediated deoxyuridine triphosphate (dUTP) nick-end-labelling (TUNEL) assay using an in situ cell death detection kit (Roche Diagnostics GmbH, Mannheim, Germany). In brief, the fixed duodenal, jejunal, and ileal segments were dehydrated, embedded in paraffin, cut into 3 μm thick sections, dewaxed, rehydrated and then incubated with 20 μg/mL proteinase K at 37 °C for 20 min. Subsequently, the sections were washed thrice with PBS before permeabilizing with 0.5% Triton X-100 at room temperature for 10 min. Next, TdT buffer containing dUTP was used to treat the sections at 37 °C for 2 h, followed by washing thrice with PBS to stop the reaction. Following this, 4′, 6-diamidino-2-phenylindole (DAPI) was used to stain the sections at room temperature for 5 min to detect cell nuclei. Finally, the sections were sealed with antifade mounting medium and imaged under a fluorescent microscope (Nikon Corporation, Tokyo, Japan). A total of six random regions were selected from each section for counting the number of TUNEL-positive cells. The apoptotic index was expressed as the proportion of apoptotic cells to total cells.

### 2.7. Western Blot Assay

Duodenal, jejunal, and ileal mucosal specimens were homogenized in RIPA buffer containing protease inhibitor cocktail (Beyotime Institute of Biotechnology, Shanghai, China) and then centrifuged at 12,000× *g* at 4 °C for 15 min to collect lysate supernatants. The total protein concentrations in the lysates were assayed using the bicinchoninic acid method [17]. The proteins were then mixed with 5× sample-loading buffer (Beyotime Institute of Biotechnology) for denaturation at 98 °C for 10 min. Thereafter, equal quantities of proteins per sample were resolved by sodium dodecyl sulphate–polyacrylamide gel electrophoresis and transferred onto polyvinylidene fluoride (PVDF) membranes. The PVDF membranes were then incubated with blocking buffer, i.e., 5% bovine serum albumin in Tris-buffered saline containing 1% Tween-20 (TBS/T), at room temperature for 1 h. After rinsing thrice with TBS/T, the PVDF membranes were probed with primary antibodies against occludin (1:1000 dilution; Abcam plc., Cambridge, UK), zonula occludens-1 (1:1000 dilution; ZO-1; Thermo Fisher Scientific, Inc., Waltham, MA, USA), or glyceraldehyde-3-phosphate dehydrogenase (1:1000 dilution; GAPDH; Cell Signalling Technology, Inc., Danvers, MA, USA) at 4 °C overnight. Following this, the PVDF membranes were rinsed thrice with TBS/T and incubated with a suitable secondary antibody at room temperature for 1 h. Finally, the PVDF membranes were immersed in Clarity™ Western ECL Substrate (Bio-Rad Laboratories, Inc., Hercules, CA, USA), visualized using a ChemiDoc™ XRS+ Imager System (Bio-Rad Laboratories, Inc.), and analyzed using ImageJ software (version 1.8.0; National Institutes of Health, Bethesda, MD, USA).

### 2.8. Statistical Analysis

All data were assessed by performing one-way analysis of variance followed by Tukey’s multiple-range tests using SAS 9.0 (SAS Inst., Inc., Cary, NC, USA), with each mouse serving as a statistical unit. The results were expressed as the means ± standard errors. *p* < 0.05 was used to denote statistical significance among means.

## 3. Results

### 3.1. Growth Performance

**Figure 1:**
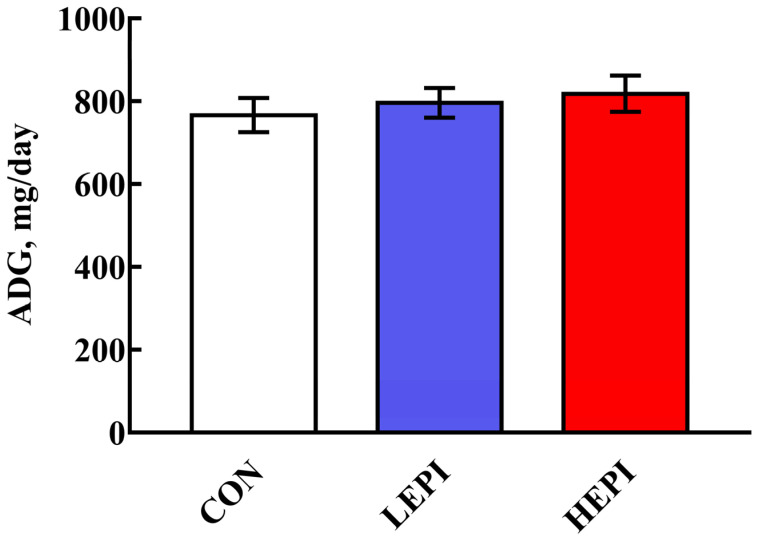
Effects of epicatechin on the growth performance of mice. Data are expressed as the means (12 mice/treatment), with standard errors represented by vertical bars. CON group: control group—mice received a standard diet; LEPI group—mice received a standard diet supplemented with 50 mg/kg epicatechin; HEPI group—mice received a standard diet supplemented with 100 mg/kg epicatechin; ADG—average daily gain.

The effects of EPI supplementation on the growth performance of the mice are presented in Figure 1. EPI supplementation did not affect (*p* > 0.05) the ADG of the mice.

### 3.2. Serum Indices

Table 1 reveals the variations in serum indices of the mice after EPI supplementation. Compared with the CON group, the serum DAO activity and _D_-lactic acid concentration were notably decreased (*p* < 0.05) in the LEPI and HEPI groups.

### 3.3. Abundance of Intestinal Tight Junction Proteins

**Figure 2:**
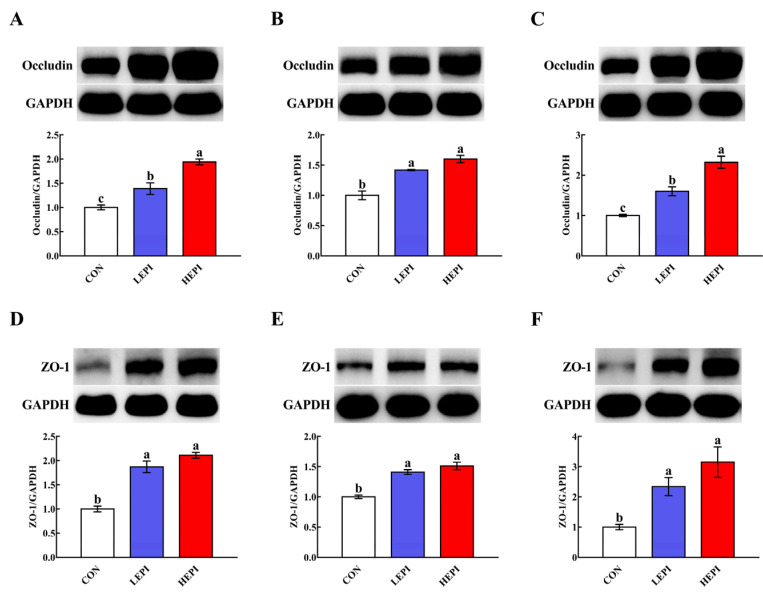
Effects of epicatechin on the abundance of intestinal tight junction proteins in mice. (**A**–**C**) Duodenal, jejunal, and ileal abundances of occludin, respectively. (**D**–**F**) Duodenal, jejunal, and ileal abundances of ZO-1, respectively. Data are expressed as means (eight mice/treatment), with standard errors represented by vertical bars. ^a–c^ Mean values with different superscripts indicate a significant difference (*p* < 0.05). CON group: control group—mice received a standard diet; LEPI group—mice received a standard diet supplemented with 50 mg/kg epicatechin; HEPI group—mice received a standard diet supplemented with 100 mg/kg epicatechin; ZO-1—zonula occludens-1; GAPDH—glyceraldehyde-3-phosphate dehydrogenase.

As shown in Figure 2, supplementation with 50 and 100 mg/kg EPI increased (*p* < 0.05) the duodenal, jejunal, and ileal abundances of occludin and ZO-1.

### 3.4. Intestinal Antioxidant Capacity

Dietary supplementation with 50 and 100 mg/kg EPI increased (*p* < 0.05) the duodenal and jejunal CAT activities and ileal SOD activity (Table 2). Additionally, supplementation with 100 mg/kg EPI increased (*p* < 0.05) the duodenal and jejunal GSH-PX activities. Moreover, supplementation with either 50 or 100 mg/kg EPI lowered (*p* < 0.05) the MDA contents in the duodenum, jejunum, and ileum.

### 3.5. Intestinal Cytokine Contents

Dietary supplementation with 50 mg/kg EPI decreased (*p* < 0.05) the ileal IL-1β content but increased (*p* < 0.05) the jejunal IL-10 content (Table 3). Moreover, dietary supplementation with 50 and 100 mg/kg EPI lowered (*p* < 0.05) the duodenal, jejunal, and ileal TNF-α contents. However, the duodenal, jejunal, and ileal IFN-γ contents remained unchanged (*p* > 0.05) after supplementation with 50 or 100 mg/kg EPI.

### 3.6. Intestinal Cell Apoptosis

**Figure 3:**
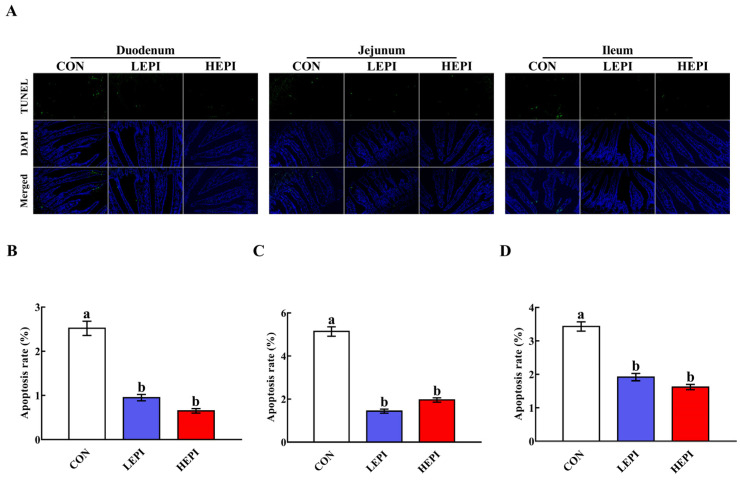
Effects of epicatechin on intestinal cell apoptosis in mice. (**A**) Representative small intestinal cross-sections of mice in the CON, LEPI, and HEPI groups after TUNEL staining (200×). Nuclei were stained with DAPI (blue) and TUNEL (green) staining. (**B**–**D**) Quantification of apoptotic cells in the duodenum, jejunum, and ileum. Data are expressed as means (eight mice/treatment), with standard errors represented by vertical bars. ^a,b^ Mean values with different superscripts indicate a significant difference (*p* < 0.05). CON group: control group—mice received a standard diet; LEPI group—mice received a standard diet supplemented with 50 mg/kg epicatechin; HEPI group—mice received a standard diet supplemented with 100 mg/kg epicatechin; DAPI—4′, 6-diamidino-2-phenylindole; TUNEL—terminal deoxynucleotidyl transferase-mediated deoxyuridine triphosphate nick-end labelling.

To assess the impacts of EPI on intestinal cell apoptosis, we observed intestinal cell apoptosis using TUNEL staining. As shown in Figure 3, 50 and 100 mg/kg EPI decreased (*p* < 0.05) duodenal, jejunal, and ileal cell apoptosis.

### 3.7. Intestinal Cell Apoptosis-Related Proteins

**Figure 4:**
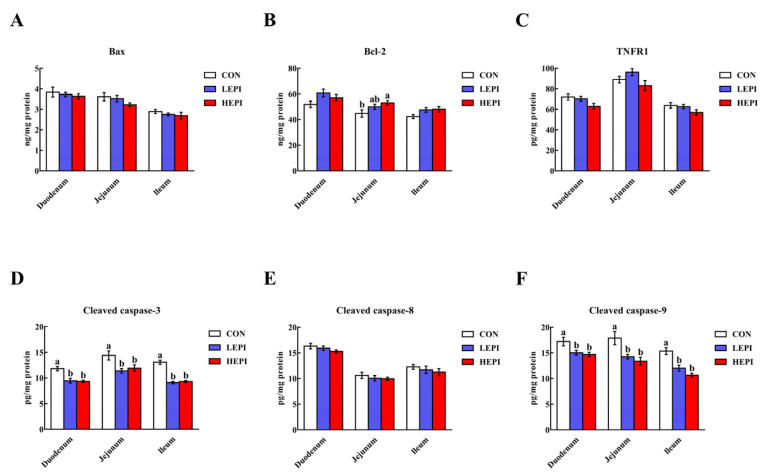
Effects of epicatechin on intestinal Bax (**A**), Bcl-2 (**B**), TNFR1 (**C**), cleaved caspase-3 (**D**), cleaved caspase-8 (**E**), and cleaved caspase-9 (**F**) contents in mice. Data are expressed as means (eight mice/treatment), with standard errors represented by vertical bars. ^a,b^ Mean values with different superscripts indicate a significant difference (*p* < 0.05). CON group: control group—mice received a standard diet; LEPI group—mice received a standard diet supplemented with 50 mg/kg epicatechin; HEPI group—mice received a standard diet supplemented with 100 mg/kg epicatechin; Bax—B-cell lymphoma-2-associated X protein; Bcl-2—B-cell lymphoma-2; TNFR1—tumor necrosis factor receptor 1; cleaved caspase-3—cleaved cysteinyl aspartate-specific proteinase-3; cleaved caspase-8—cleaved cysteinyl aspartate-specific proteinase-8; cleaved caspase-9—cleaved cysteinyl aspartate-specific proteinase-9.

Supplementation with 50 and 100 mg/kg EPI decreased (*p* < 0.05) the cleaved caspase-3 and cleaved caspase-9 contents in the duodenum, jejunum, and ileum (Figure 4). In contrast, supplementation with 100 mg/kg EPI increased (*p* < 0.05) the jejunal Bcl-2 content. However, no changes (*p* > 0.05) were noted in the duodenal, jejunal, and ileal Bax, TNFR1, and cleaved caspase-8 contents in the CON, LEPI, and HEPI groups.

## 4. Discussion

The intestinal barrier is closely related to tight junctions, which are dynamic multifunctional complexes that are located between epithelial cells and function as a critical structure for paracellular permeability [18]. Tight junctions consist of transmembrane proteins, such as occludin, and cytosolic proteins that are recruited to the apicolateral membrane, such as ZO-1 [19,20]. We found that EPI supplementation increased the duodenal, jejunal, and ileal abundances of occludin and ZO-1 (at 50 and 100 mg/kg). These findings indicate that EPI supplementation could improve intestinal barrier integrity in mice. At present, several blood parameters, such as DAO activity and _D_-lactic acid concentration, are considered circulating markers for evaluating the degree of intestinal barrier damage. Intestinal barrier disruption usually leads to the release of DAO and _D_-lactic acid into blood circulation [21,22]. Accordingly, mice treated with EPI (50 and 100 mg/kg) exhibited a decrease in serum DAO activity and _D_-lactic acid concentration, further confirming that EPI supplementation could improve the intestinal barrier integrity in mice.

In addition to playing a crucial role in the modulation of intestinal inflammatory responses, cytokines (both pro-inflammatory and anti-inflammatory cytokines) have an important effect on the regulation of intestinal barrier integrity [23,24]. Most pro-inflammatory cytokines, such as IL-1β and TNF-α, can disrupt the intestinal barrier, thereby increasing intestinal permeability [25,26]. We found that supplementation with 50 mg/kg EPI decreased the ileal IL-1β content, and supplementation with 50 and 100 mg/kg EPI decreased the duodenal, jejunal, and ileal TNF-α contents in mice. These findings indicate that EPI supplementation could improve intestinal barrier integrity by suppressing the release of intestinal pro-inflammatory cytokines. On the other hand, IL-10, an anti-inflammatory cytokine, can attenuate intestinal inflammatory injury [27]. We observed that supplementation with 50 mg/kg EPI increased the jejunal IL-10 content in mice. Intriguingly, EPI supplementation may also improve intestinal barrier integrity by promoting the production of intestinal anti-inflammatory cytokines.

Another pivotal factor contributing to intestinal barrier disruption is oxidative stress, which is widely recognized as a state of imbalance between oxidation and antioxidation [28]. MDA is the primary product of lipid peroxidation and is usually considered an oxidative stress marker [29,30]. We noted a decrease in duodenal, jejunal, and ileal MDA contents in mice treated with EPI (50 and 100 mg/kg), indicating that intestinal oxidative damage was attenuated by EPI supplementation. The activities of antioxidant enzymes, including SOD, CAT, and GSH-PX, are important indicators of antioxidant capacity. SOD catalyzes the conversion of superoxide into hydrogen peroxide, and CAT and GSH-PX convert hydrogen peroxide into water [31,32]. Importantly, we found that supplementation with 50 and 100 mg/kg EPI increased the duodenal and jejunal CAT activities and ileal SOD activity, and supplementation with 100 mg/kg EPI increased the duodenal and jejunal GSH-PX activities in mice. These results suggest that EPI could alleviate intestinal oxidative damage by enhancing antioxidant enzyme activities in the intestines of mice, thereby improving intestinal barrier integrity.

Although cell apoptosis is essential for intestinal epithelial turnover and tissue homeostasis, excessive apoptosis can result in intestinal barrier damage [33]. Therefore, the reduced duodenal, jejunal, and ileal cell apoptosis noted in our study after supplementation with 50 and 100 mg/kg EPI indicates that EPI could improve intestinal barrier integrity in mice through the inhibition of apoptosis. In general, cell apoptosis occurs via the intrinsic pathway (mitochondria-dependent pathway) or the extrinsic pathway (death-receptor-dependent pathway) [34]. The intrinsic pathway is regulated by the Bcl-2 family and is mainly characterized by caspase-9 activation [35,36], while the extrinsic pathway is mediated by membrane death receptors, such as TNFR1, and is mainly characterized by caspase-8 activation [37,38]. Caspase-3 is an executioner caspase that is activated by these two pathways and then initiates apoptosis [39,40]. We found that dietary supplementation with 50 and 100 mg/kg EPI decreased cleaved caspase-3 and cleaved caspase-9 contents in the duodenum, jejunum, and ileum of mice. These results indicate that EPI could repress intestinal cell apoptosis in mice in a mitochondria-dependent manner.

## 5. Conclusions

In summary, our findings indicate that EPI was beneficial in enhancing intestinal barrier integrity in mice. The underlying mechanisms may be closely related to the suppression of intestinal inflammation and oxidative stress and the reduction in cell apoptosis. These findings provide a foundation for developing EPI as a nutraceutical to improve intestinal health.

## Figures and Tables

**Table 1:** Effects of epicatechin on the serum indices of mice ^†^.

Items ^§^	Treatment Groups ^‡^			*p*-Value
	CON	LEPI	HEPI	
DAO, U/L	9.04 ± 0.25 ^a^	7.48 ± 0.14 ^b^	7.27 ± 0.16 ^b^	<0.001
_D_-Lactic acid, ng/mL	563.64 ± 8.17 ^a^	488.49 ± 5.73 ^b^	507.46 ± 4.32 ^b^	<0.001

^a,b^ Mean values with different superscripts in the same row indicate a significant difference (*p* < 0.05). ^†^ Values are the means of eight replicates per treatment. ^‡^ CON group: control group—mice received a standard diet; LEPI group—mice received a standard diet supplemented with 50 mg/kg epicatechin; HEPI group—mice received a standard diet supplemented with 100 mg/kg epicatechin. ^§^ DAO—diamine oxidase.

**Table 2:** Effects of epicatechin on the intestinal antioxidant capacity of mice ^†^.

Items ^§^	Treatment Groups ^‡^			*p*-Value
	CON	LEPI	HEPI	
Duodenum				
SOD, U/mg protein	70.90 ± 2.89	81.74 ± 3.50	79.20 ± 4.66	0.127
CAT, U/mg protein	46.56 ± 2.48 ^c^	66.33 ± 2.88 ^b^	78.54 ± 2.79 ^a^	<0.001
GSH-PX, U/mg protein	184.44 ± 4.55 ^b^	201.16 ± 4.75 ^b^	241.52 ± 7.83 ^a^	<0.001
MDA, nmol/mg protein	2.97 ± 0.18 ^a^	1.66 ± 0.09 ^b^	1.47 ± 0.10 ^b^	<0.001
Jejunum				
SOD, U/mg protein	50.14 ± 1.88	53.99 ± 2.62	51.10 ± 2.15	0.461
CAT, U/mg protein	23.26 ± 0.91 ^b^	29.07 ± 1.85 ^a^	30.65 ± 1.58 ^a^	0.006
GSH-PX, U/mg protein	170.46 ± 4.85 ^b^	180.83 ± 3.37 ^a, b^	185.56 ± 3.79 ^a^	0.044
MDA, nmol/mg protein	1.97 ± 0.08 ^a^	1.53 ± 0.07 ^b^	1.38 ± 0.04 ^b^	<0.001
Ileum				
SOD, U/mg protein	54.23 ± 2.76 ^b^	71.78 ± 2.24 ^a^	80.04 ± 2.60 ^a^	<0.001
CAT, U/mg protein	29.24 ± 1.29	30.53 ± 1.50	33.89 ± 1.88	0.123
GSH-PX, U/mg protein	127.92 ± 3.85	138.81 ± 5.06	141.77 ± 4.47	0.095
MDA, nmol/mg protein	2.69 ± 0.09 ^a^	2.11 ± 0.10 ^b^	2.01 ± 0.07 ^b^	<0.001

^a–c^ Mean values with different superscripts in the same row indicate a significant difference (*p* < 0.05). ^†^ Values are the means of eight replicates per treatment. ^‡^ CON group: control group—mice received a standard diet; LEPI group—mice received a standard diet supplemented with 50 mg/kg epicatechin; HEPI group—mice received a standard diet supplemented with 100 mg/kg epicatechin. ^§^ SOD—superoxide dismutase; CAT—catalase; GSH-PX—glutathione peroxidase; MDA—malondialdehyde.

**Table 3:** Effects of epicatechin on the intestinal cytokine contents of mice ^†^.

Items ^§^	Treatment Groups ^‡^			*p*-Value
	CON	LEPI	HEPI	
Duodenum, pg/mg protein				
IL-1β	30.85 ± 1.38	32.75 ± 1.23	28.47 ± 1.13	0.075
IL-10	486.92 ± 12.63	517.91 ± 16.42	497.73 ± 12.89	0.308
TNF-α	309.87 ±7.39 ^a^	230.81 ± 5.28 ^b^	212.71 ± 9.55 ^b^	<0.001
IFN-γ	338.01 ± 9.43	324.94 ± 7.32	319.80 ± 8.59	0.315
Jejunum, pg/mg protein				
IL-1β	32.68 ± 1.18	30.68 ± 0.80	30.31 ± 0.85	0.193
IL-10	440.39 ± 11.10 ^b^	535.18 ± 8.24 ^a^	466.93 ± 14.38 ^b^	<0.001
TNF-α	325.84 ± 7.47 ^a^	301.07 ± 6.86 ^b^	297.19 ± 6.17 ^b^	0.015
IFN-γ	310.94 ± 6.12	301.32 ± 7.30	294.26 ± 6.78	0.238
Ileum, pg/mg protein				
IL-1β	27.69 ± 1.32 ^a^	22.74 ± 0.57 ^b^	25.46 ± 1.12 ^a,b^	0.011
IL-10	351.85 ± 12.71	353.51 ± 10.48	380.03 ± 9.19	0.147
TNF-α	231.54 ± 6.88 ^a^	203.52 ± 5.97 ^b^	194.74 ± 9.31 ^b^	0.006
IFN-γ	240.08 ± 11.15	225.44 ± 8.35	234.89 ± 5.88	0.497

^a,b^ Mean values with different superscripts in the same row indicate a significant difference (*p* < 0.05). ^†^ Values are the means of eight replicates per treatment. ^‡^ CON group: control group—mice received a standard diet; LEPI group—mice received a standard diet supplemented with 50 mg/kg epicatechin; HEPI group—mice received a standard diet supplemented with 100 mg/kg epicatechin. ^§^ IL-1β—interleukin-1β; IL-10—interleukin-10; TNF-α—tumor necrosis factor-α; IFN-γ—interferon-γ.

## Data Availability

All data presented in this study are available through the corresponding author.
